# Correlative infrared nanospectroscopic and nanomechanical imaging of block copolymer microdomains

**DOI:** 10.3762/bjnano.7.53

**Published:** 2016-04-22

**Authors:** Benjamin Pollard, Markus B Raschke

**Affiliations:** 1 Department of Physics, Department of Chemistry, and JILA, University of Colorado, Boulder, Colorado 80309, USA

**Keywords:** block copolymers, force–distance nanomechanical spectroscopy, hybrid imaging, near-field infrared spectroscopy, scanning probe microscopy

## Abstract

Intermolecular interactions and nanoscale phase separation govern the properties of many molecular soft-matter systems. Here, we combine infrared vibrational scattering scanning near-field optical microscopy (IR *s*-SNOM) with force–distance spectroscopy for simultaneous characterization of both nanoscale optical and nanomechanical molecular properties through hybrid imaging. The resulting multichannel images and correlative analysis of chemical composition, spectral IR line shape, modulus, adhesion, deformation, and dissipation acquired for a thin film of a nanophase separated block copolymer (PS-*b*-PMMA) reveal complex structural variations, in particular at domain interfaces, not resolved in any individual signal channel alone. These variations suggest that regions of multicomponent chemical composition, such as the interfacial mixing regions between microdomains, are correlated with high spatial heterogeneity in nanoscale material properties.

## Introduction

Functional soft-matter and polymer systems often exhibit novel phenomena due to nanoscale chemical heterogeneity and the resulting intermolecular interactions. Infrared vibrational scattering scanning near-field optical microscopy (IR *s*-SNOM) provides a direct, noninvasive, label-free measure of nanoscale chemical composition by localizing the light–matter interaction via a scanning probe tip [1]. Performed most simply with a single-frequency source tuned to a molecular marker resonance [2], IR *s*-SNOM also enables IR spectroscopy on the nanoscale using broadband [3–4] or tunable light sources [5]. Combined with computational imaging to analyze spectral peak position and lineshape, as well as polarization selection, *s*-SNOM can probe intermolecular coupling [6], polymorphism [7], molecular orientation, domain structure [8], and degrees of crystallinity.

However, information beyond nanoscale IR response is sometimes desired for a more complete understanding of molecular interactions and their relationship to material function. Nanomechanical properties, measured through force spectroscopy, can provide important complimentary information on heterogeneous material systems [9]. By measuring the force on a scanning probe tip as it interacts with the sample, material properties including friction, adhesion, deformation, modulus, and dissipation can be quantified and mapped over nanoscale distances [10–14].

Here, we combine IR *s*-SNOM and force–distance spectroscopy for a multimodal study of heterogeneous molecular thin films. Although *s*-SNOM is commonly already based on intermittent-contact atomic force microscopy (AFM), the enabled compatibility with numerous advanced scanning probe modalities, including force–distance spectroscopy, has not yet been explored in combination with *s*-SNOM. We perform both spatio-spectral *s*-SNOM and force–distance nanoimaging and nanospectroscopy on mesoscopic regions of a block copolymer, providing a multidimensional picture of the variation in material and optical properties between and within polymer microdomains.

## Methods

We study a high molecular weight block copolymer of polystyrene-*block*-poly(methyl methacrylate) (PS-*b*-PMMA), with a relative chain length of 270.0-*b*-289.0 *M*_n_ × 10^3^ (P4443-SMMA, Polymer Source), spin-coated from a 1% w/v solution in toluene onto native-oxide silicon substrates (2 kRPM). The film thickness is *~*60 nm, as measured with AFM. The PMMA in this sample is mostly syndiotactic. Long chain lengths result in nonequilibrium, irregularly shaped, microdomain structures with incomplete microphase separation and large interfacial mixing regions [6,15–16]. The size of these microdomains (*~*60 nm, determined by the radial peak of a 2D Fourier transform of AFM topography) is comparable to the film thickness. Towards equilibrium, differences in the surface energy of PS and PMMA lead to the formation of a top surface layer of PS at the air interface, as PMMA is more polar than PS and tends toward the polar SiO_2_ native oxide layer of the Si substrate [17]. Models suggest that this PS surface layer is roughly half the microdomain size when in equilibrium [18], though we can expect nonuniformity and local variations of the surface layer in this case due to the long chain lengths.

We measure the nanomechanical properties of the copolymer film using several modes of scanning force microscopy. We use intermittent contact mode under ambient conditions to map the ordering of block copolymer domains [19]. This modality, especially its phase images, is sensitive to the viscoelastic properties of the sample [20]. To further quantify nanoscale material properties, we also use force–distance spectroscopy (peak force quantitative nanomechanical mapping, PF-QNM) to map spatial variations in modulus, as well as the adhesion, deformation, and dissipation of the tip–sample interaction, simultaneously [12–13]. Corresponding quantitative values can be extracted from calibrated force–distance curves at every pixel to build up a multidimensional force–distance image.

**Figure 1:**
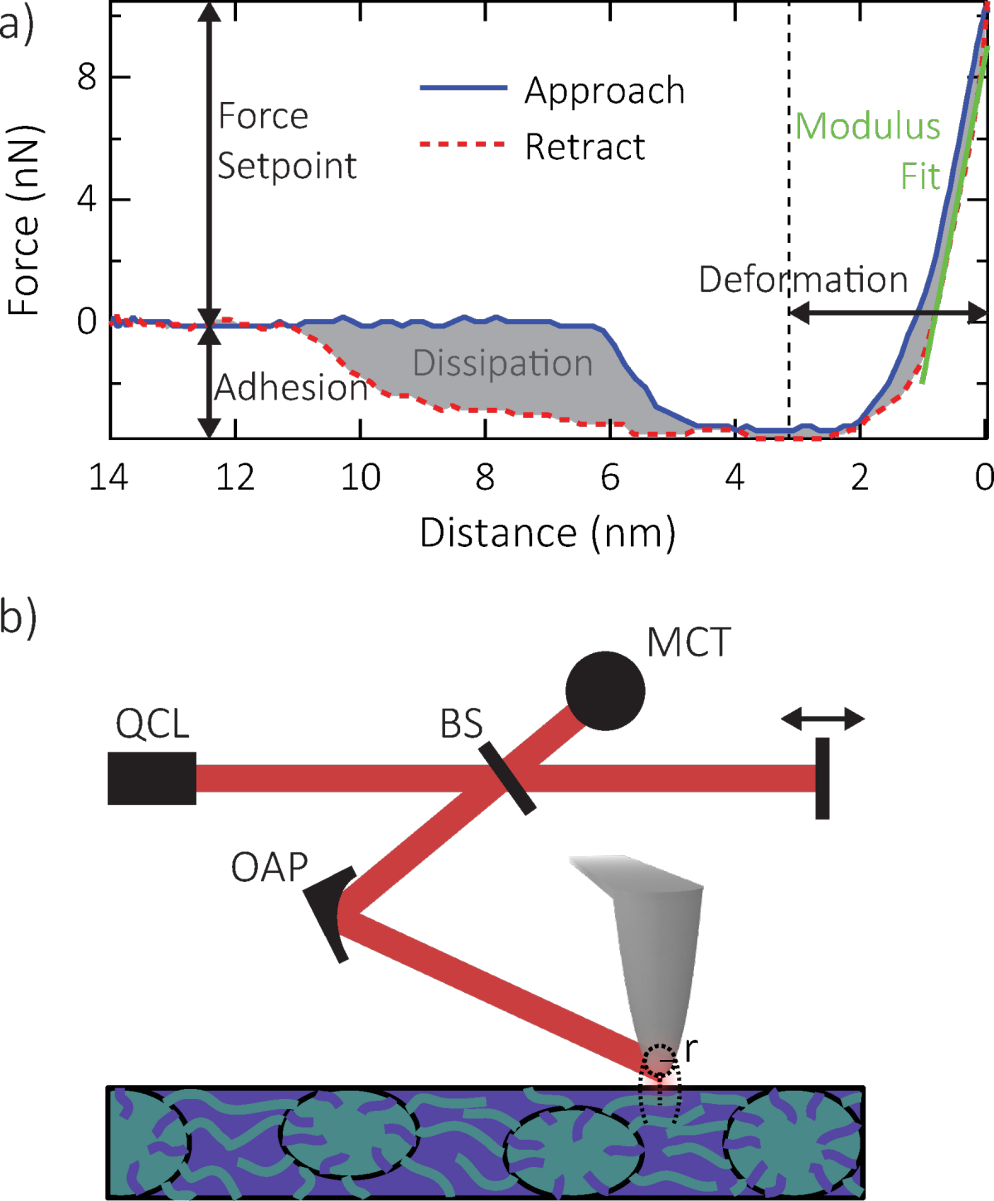
(a) Example force–distance curve from a single force volume at a given force setpoint, showing the properties that are analyzed to create maps of modulus, adhesion, deformation, and dissipation. (b) Schematic of *s*-SNOM setup, showing the quantum cascade laser (QCL), beam splitter (BS), off-axis parabolic (OAP) mirror, mercury–cadmium–telluride (MCT) detector, moving reference arm mirror, and tip–sample interaction confined to the tip radius *r*. Sample feature size exaggerated for visual clarity.

In this work we acquire force–distance curves, as shown in Figure 1a, using a modified commercial AFM (MultiMode 8, Bruker). We calibrate quantiative force values using measurements with the same tip on a Si sample and a rough TiO_2_ surface for measuring the deflection sensitivity and tip radius, respectively, as well as tuning curves for measuring the Q-factor using the Sader method [21]. We use commercial Tap150 and Tap525 tips (Bruker) for initial PF-QNM measurements (not shown), and then use those values to calibrate PF-QNM data taken with metallized tips as necessary for *s*-SNOM. Note that the curve in Figure 1a was taken with a different tip and slightly different scan settings than the calibrated data shown below; the curve here is shown only to illustrate the different PF-QNM channels.

In IR *s*-SNOM, conventional AFM is combined with an optical setup to focus and detect light from a tip-mediated near-field optical interaction confined to a nanoscale volume of the sample surface, as shown schematically in Figure 1b. The localized tip–sample interaction depends on the optical properties of the sample directly below the apex. By scanning the sample, keeping the tip stationary with respect to the laser focus, we can create maps of optical properties of the sample simultaneously with AFM data channels [1].

For our *s*-SNOM experiments, we use a metallized scanning probe tip (11.72 N/m, PtSi–NCH, NanoWorld AG). Mid-infrared light tunable between 1660–1900 cm^−1^ from a quantum cascade laser (QCL, Daylight Solutions) is focused onto the tip, linearly polarized along the surface normal relative to the sample, using an off-axis parabolic mirror (NA = 0.45) with a power density of ≤50 MW cm^−2^. Tip-scattered light is detected in a confocal epi-illumination/detection geometry with a LN_2_-cooled HgCdTe detector (MCT, KLD-0.25/DC/11.5, Kolmar Technologies). The far-field background is suppressed by a lock-in detector (HF2, Zurich Instruments) demodulating at the third harmonic of the tip tapping frequency. Tip-scattered light is recombined at the detector with light of known phase from the reference arm in an asymmetric Michelson interferometer geometry, allowing for the determination of spectral amplitude 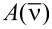 and phase 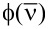 of the near-field signal [22].

The vertical sensitivity of *s*-SNOM falls off rapidly into the film over the near-field decay length, which is given by the tip radius to first order [23]. Thus, the probed region penetrates at least in part through a possible nonresonant PS surface layer. Therefore, resonant near-field phase contrast reveals the presence of PMMA microdomains.

**Figure 2:**
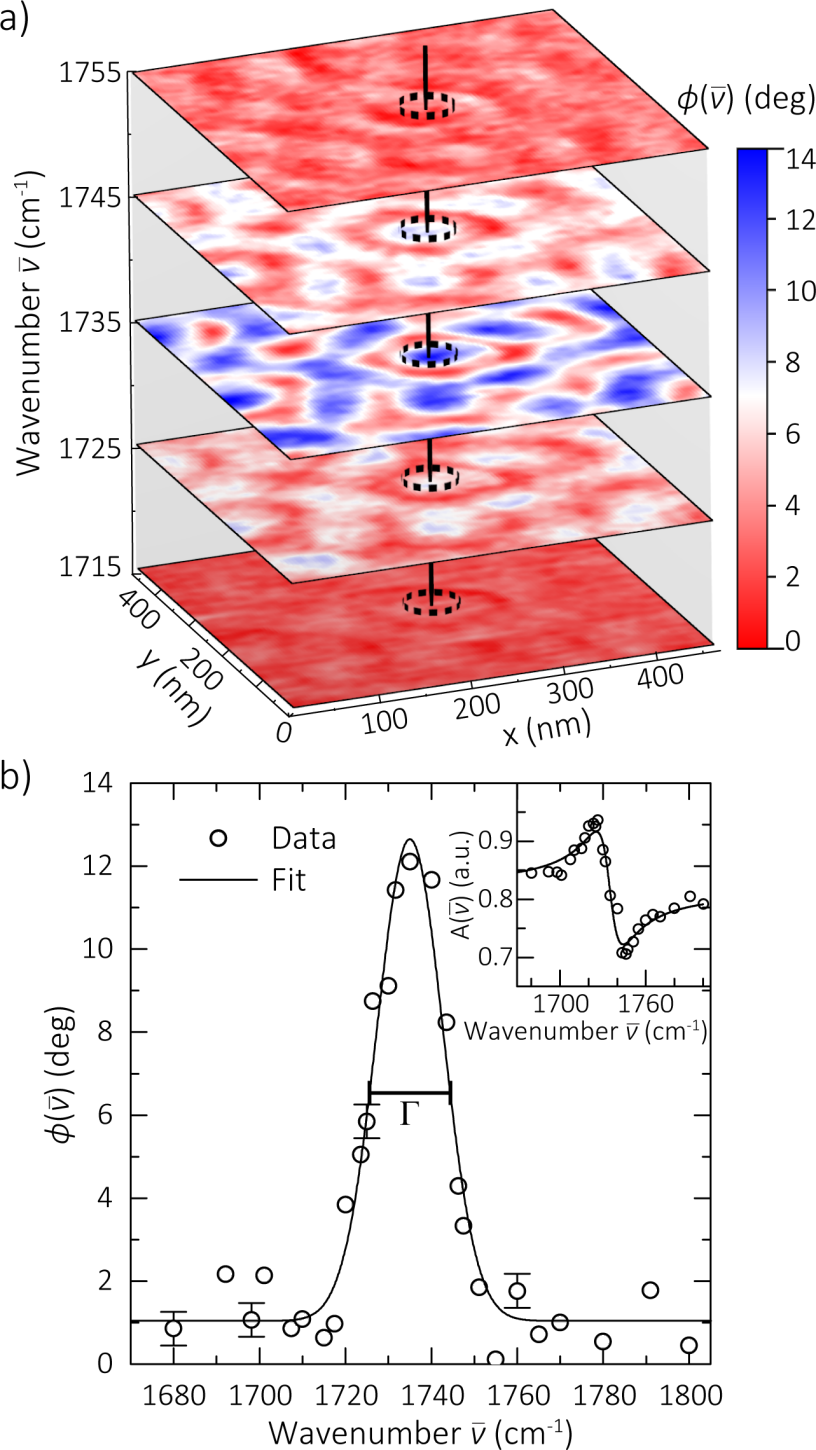
(a) Spatio-spectral *s*-SNOM phase image of PS-*b*-PMMA, with carbonyl mode resonant contrast between PMMA (blue) and PS (red) microdomains. (b) Spectrum extracted from a single PMMA microdomain, as illustrated by the vertical line in (a). Error bars shown on select points as reference, calculated as the standard deviation of the nonresonant baseline datapoints. Peak position and linewidth values Γ are extracted computationally from Gaussian fits to spectra at each pixel. Corresponding amplitude spectrum (inset) also shown for completeness, with fit to a dispersive lineshape as a guide to the eye.

A spatio-spectral *s*-SNOM map is acquired over a 500 × 500 nm size region of PS-*b*-PMMA, shown in Figure 2a, as previously described [5–6]. Images are taken over a range of wavelengths spanning the carbonyl stretch mode. The images are 128 × 128 pixels acquired at a rate of 0.3 Hz per line, resulting in a pixel size of 4 × 4 nm and total acquisition time of *~*7 min per image. The set of images is analyzed to create nanoscale IR spectra from individual microdomains, as illustrated in Figure 2b. We use the *s*-SNOM spectral phase 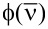 in our analysis, which for weak molecular oscillators provides a good approximation of the spectral shape of the optical absorption or extinction coefficient 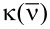
[1]. Gaussian resonance curves are computationally fit to the spectra in order to extract peak position 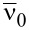 and linewidth Γ based on

[1]
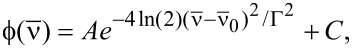


where *A* and *C* are frequency-independent scaling and offset constants and Γ is the full width at half maximum (FWHM). For example, the corresponding fit shown in Figure 2b yields a peak position of 1735 ± 1 cm^−1^ and Γ of 18 ± 2 cm^−1^, with the margin of error representing the 95% confidence interval of the fit parameter. While Lorentzian lineshape fits (not shown) yield similar results, Gaussian lineshapes fit the data slightly better, which indicates that the probed oscillator ensemble is dominated by inhomogeneous broadening. The corresponding amplitude spectrum is shown in the inset with calculated lineshape (solid line) based on the above fit parameters and is Kramers–Kronig consistent.

As is often encountered in hybrid imaging, maps from force–distance spectroscopy and from *s*-SNOM acquired subsequently with the same tip need to be aligned to compensate for sample drift. The scans are registered by visually comparing *~*10 like features in the height images common to both techniques. The different image datasets are then aligned, with few nanometer accuracy, using standard image registration techniques and an affine transform (MATLAB Image Processing Toolbox).

## Results and Discussion

**Figure 3:**
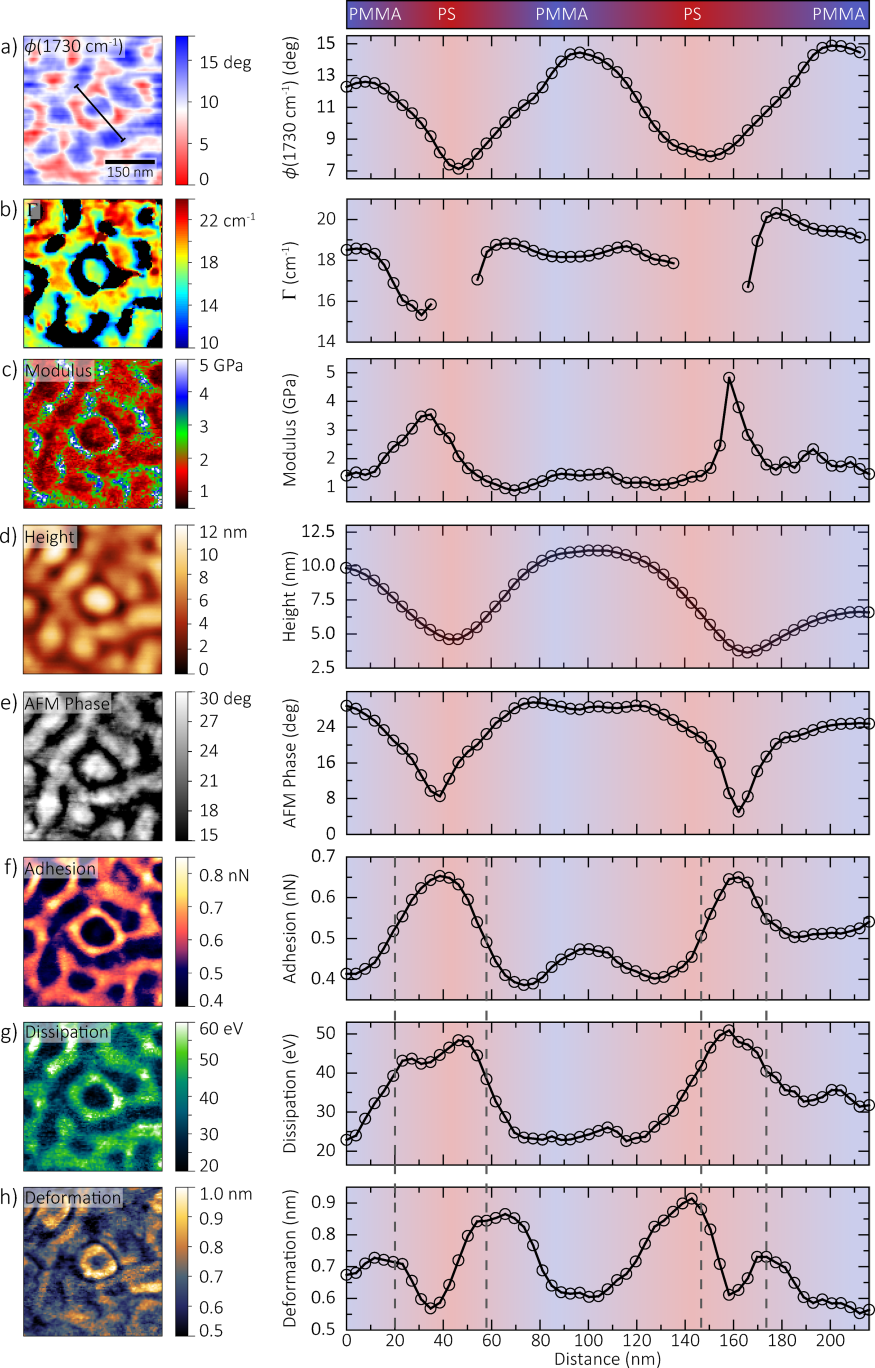
Multidimensional dataset showing maps of PS and PMMA microdomains, with corresponding profiles along the line indicated in (a) averaged over a 5-pixel width. Color bar and profile background suggest regions of high PS or PMMA concentration. (a,b,d,e) measured using spatio-spectral *s*-SNOM and (c,f–h) measured using force–distance spectroscopy. Differences between channels, as highlighted by the dashed lines in (f–h), indicate a complex interplay between crystallinity, composition, and intermolecular interaction between and within single domains.

Combined *s*-SNOM and force–distance images of a 500 × 500 nm size region of PS-*b*-PMMA are shown in Figure 3 with individual PS and PMMA microdomains and/or surface variations clearly resolved in all imaging channels. The microdomains are *~*60 nm in size, and finer features are observed in some channels with an overall spatial resolution of 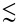
20 nm. The *s*-SNOM phase in Figure 3a measured on-resonance of the carbonyl stretch mode at 1730 cm^−1^ reflects the nanoscale surface chemical composition. The corresponding trace to the right shows the indicated line profile averaged over a 5-pixel width. The profile starts and ends in a PMMA-rich region and passes through a microdomain surrounded by PS, as indicated by the color bar and profile background. The *s*-SNOM image in Figure 3a is one of many scans in the spatio-spectral dataset used to create the linewidth map shown in Figure 3b. The black regions in Figure 3b represent PS-rich regions where there is insufficient resonant PMMA signal to perform a computational fit and extract a linewidth value. Standard intermittent contact mode topography (Figure 3d) and tapping phase (Figure 3e) channels were collected concurrently during *s*-SNOM scans. The images of modulus (Figure 3c), adhesion (Figure 3f), deformation (Figure 3g), and dissipation (Figure 3h) are defined from the analysis of force–distance curves taken with the same tip over the same region, calibrated as described above.

We present here a preliminary discussion of the polymer properties and interactions probed by our hybrid imaging technique as an illustration of the information made available, while also recognizing that understanding the full complexity of the material system requires more detailed measurements and analysis.

In the resonant *s*-SNOM image (Figure 3a), carbonyl groups in PMMA provide a marker resonance at *~*1730 cm^−1^, resulting in a measured *s*-SNOM phase contrast on the order of 10° compared to the nonresonant PS regions. Within apparent PMMA microdomains, linewidths (Figure 3b) varied between 15 to 20 cm^−1^. The carbonyl resonance is sensitive to the local chemical environment through intermolcular interactions, resulting in vibrational solvatochromism and line broadening [6]. The varying concentrations of neighboring carbonyl groups in adjacent MMA monomers result in differing levels of intermolecular coupling, changing the measured nanoscale spectral linewidth. In addition, anisotropy within a single PMMA region itself could affect the degree of intermolecular coupling, manifesting in linewidth changes within a single microdomain.

The modulus image (Figure 3c) shows contrast from the differing mechanical properties of PMMA and PS. We observe values around 1–2 GPa in PMMA regions, and 3–4 GPa in PS regions. These values of the DMT modulus [24] are closely related to the Young’s modulus, an intrinsic bulk material property of the sample. Measurements of Young’s modulus in polymers often exhibit considerable variation and nonlinearity [25]. However, our values generally match the range measured on PS-*b*-PMMA from similar techniques [14,26–27].

The height (Figure 3d), tapping phase (Figure 3e), adhesion (Figure 3f), dissipation (Figure 3g), and deformation (Figure 3h) images represent aspects of the physical tip–sample interaction. For example, we observe a topographic height difference in Figure 3d of *~*5 nm between PMMA and PS regions, with PMMA appearing higher, while the tapping phase in Figure 3e is *~*15° larger for PMMA than PS. In this case, slight differences in the presence of residual toluene solvent between PMMA and PS, with PMMA retaining slightly more, likely results in PS microdomains shrinking slightly more than PMMA microdomains during spin casting and subsequent drying [28].

The tapping phase is related to the power dissipated by the sample during contact with the tip, and reflects to some extent the viscoelastic properties of the sample [20]. However, the tapping phase is also affected by the intermittent formation of a capillary water neck between tip and sample as the cantilever oscillates, which can lead to either net attractive or net repulsive regimes depending on tapping amplitude, relative humidity, and local curvature of the tip and sample [29–30]. Modeling the formation, evolution, and breaking of the tip–sample water menisci in order to understand the effect on the tapping phase is still a focus of active research [31].

We measure an overall adhesion (Figure 3f) of 0.4 nN on PMMA, and 0.7 nN on PS. We also observe finer structure within regions in the corresponding profile, with an increase of around 0.1 nN in the center of the PMMA microdomain compared to the edges. The adhesion channel measures the minimum force during the force–distance curve, which is sensitive to attractive forces between tip and sample (e.g., electrostatic or capillary forces). It can reflect variations in the Hamaker constant of the van der Waals interaction, surface charges, or hydrophilicity [32]. It has been observed in PS-*b*-PMMA that PS preferentially adsorbs onto a gold surface compared to PMMA [33]. Thus, the higher attractive forces over PS domains suggest that our metallized tip interacts with PS similarly to such chemically inert gold substrates. In addition, the polar nature of PMMA makes it more hydrophilic than pure PS [34]. Thus in our block copolymer, the lower attractive forces over PMMA domains possibly indicate that we are operating in the repulsive capillary force regime. Tip–surface capillary forces are most studied in the context of resonant cantilever motion instead of the slower, nonresonant distance modulation employed in PF-QNM. Nonetheless, our modulation amplitude (15 nm), measured tip radius (16–25 nm), and the relative humidity during the measurement (13%) indicate that we are operating near the threshold between attractive and repulsive regimes [30]. The delicate balance between repulsive capillary forces and overall van der Waals interaction could result in the finer variations across the PMMA microdomain.

We measure a dissipation (Figure 3g) of 25 eV over PMMA, and 45 eV over PS. The dissipation channel measures the integrated hysteresis between approach and retract and thus directly measures the energy lost to the sample. For purely elastic behavior, the energy loss is dominated by adhesive forces associated with the tip–surface interaction, while for inelastic deformation, energy is transferred into the sample itself [32] or to the surface water layer through the formation and rupture of the capillary neck at different distances [30]. In the case of PS-*b*-PMMA, the positive correlation between dissipation and adhesion suggests that energy loss is dominated by surface-sensitive adhesive forces.

Our deformation map (Figure 3h) does not correlate directly with chemical composition (e.g., dissipation), as indicated by the dotted lines in the corresponding profile. Instead, it shows larger values, 0.9 nm, at the interfaces between the PS and PMMA domains, compared to 0.6 nm at the domain centers. The deformation measures the maximum penetration of the tip into the polymer during the force–distance cycle, which can be due to both elastic and inelastic behavior, and thus is a measure of both the elasticity and the hardness of the sample [32]. Polymers, including syndiotactic PMMA, tend to pack in a semi-ordered way to minimize the total free energy, though this is highly dependent on tacticity and molecular weight distribution [35]. Crystallinity also creates varying mechanical properties, as greater order and coupling generally suggest stiffer regions at the center of domains [25]. Our deformation profile suggests that the mixing of PMMA and PS significantly disrupts the polymer order and softens the interfacial region.

**Figure 4:**
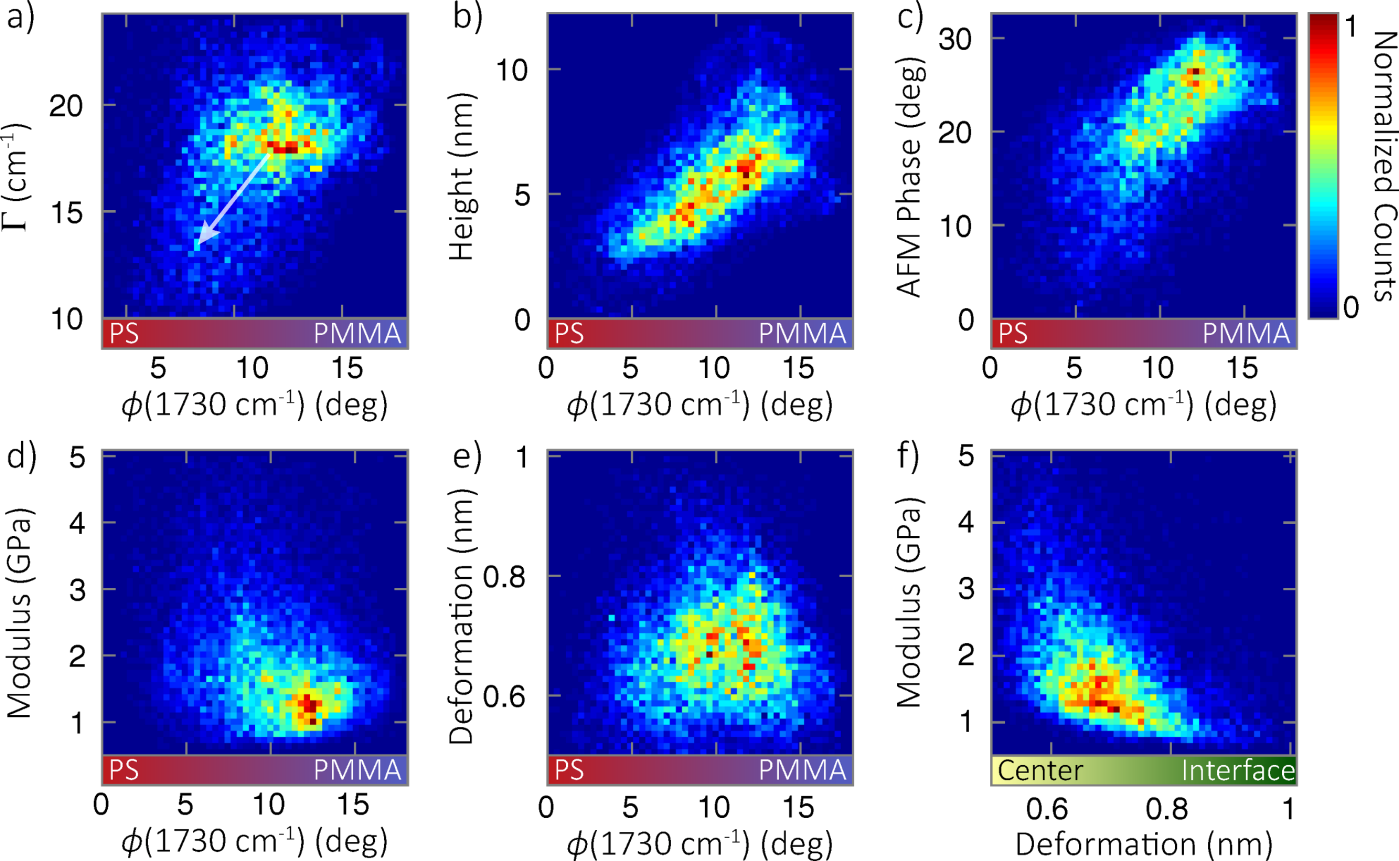
Bivariate histograms, showing the correlations between different signal channels across the entire image region. The arrow in (a) indicates that the correlation is seen to continue towards the lower left until Γ becomes undefined in nonresonant, PS-rich regions. Colored bars along the horizontal axes illustrate the correspondence of the spectral phase with PMMA concentration, or deformation with interfacial mixing (center vs interface).

To further illustrate the relationships between the different image channels, Figure 4 shows several bivariate histograms displaying correlations between two respective channels across the entire image region. They confirm the trends discussed above, as Figure 4a–c show a clear positive correlation of resonant *s*-SNOM phase with Γ, height, and AFM phase, illustrating that these channels are predominantly sensitive to differences in relative chemical concentration of PS and PMMA. Note in Figure 4a that Γ is not defined in nonresonant PS regions. However, the correlation with 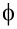
(1730 cm^−1^) is seen to continue, with decreasing Γ as the PMMA is diluted in the PS phase, and is consistent with earlier results [6]. This relationship is indicated by the arrow in Figure 4a. Modulus and deformation, however, show little correlation with 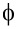 (Figure 4d,e), instead showing a negative correlation with each other (Figure 4f). Those channels are predominantly sensitive to differences in interfacial mixing between microdomains, suggesting a complex interplay between crystallinity, composition, and intermolecular interactions, especially at microdomain interfaces.

**Figure 5:**
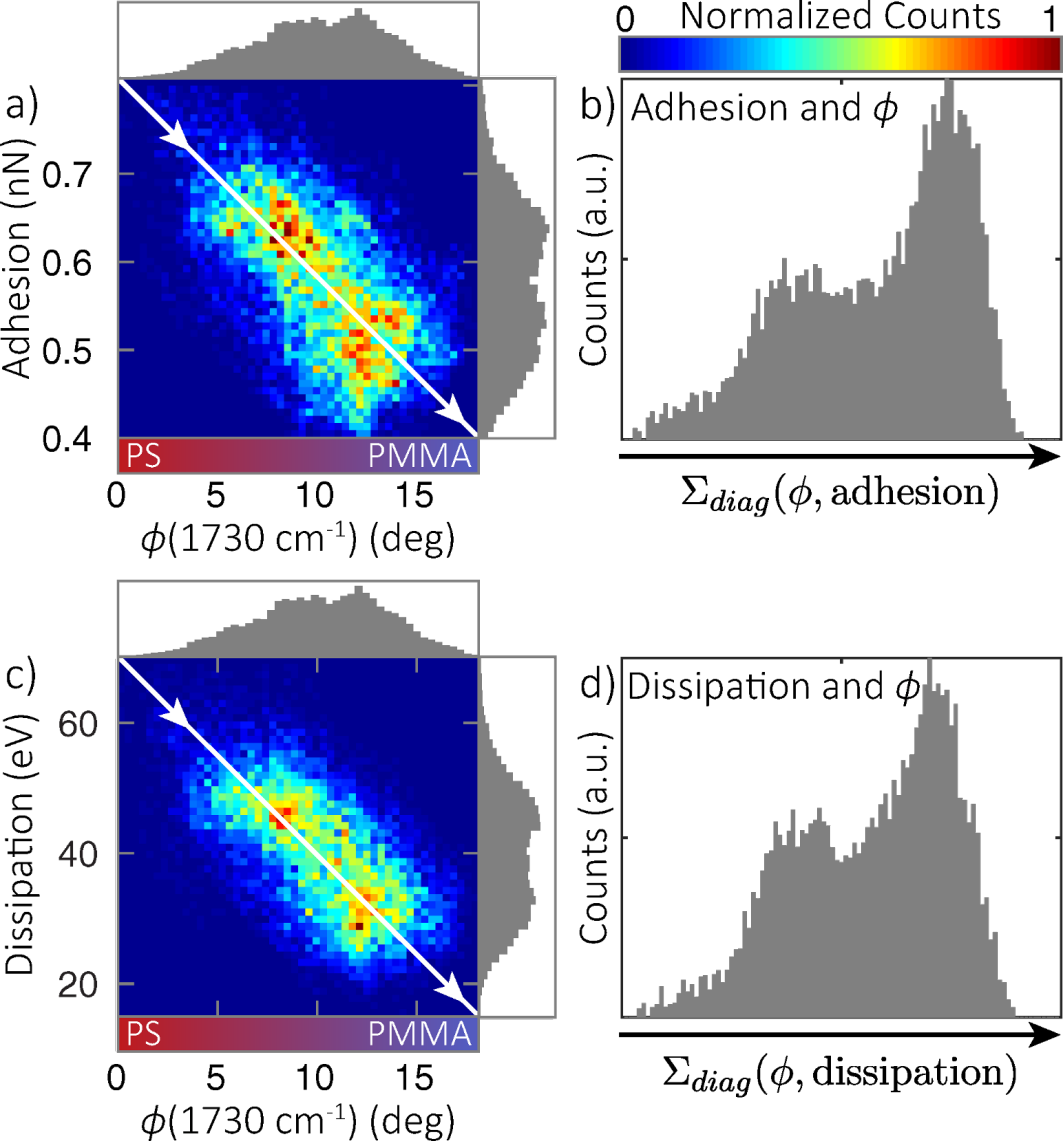
Bivariate and corresponding coincidence histograms showing correlations between adhesion and 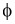
(1730 cm^−1^) (a,b), and dissipation and 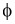
(1730 cm^−1^) (c,d). Histograms of each single channel are shown along the corresponding axis in (a) and (c). Coincidence histograms (b,d) calculated by summing counts in the bivariate histograms (a,c) along diagonal lines perpendicular to the direction of correlation (solid line). A bimodal distribution is apparent, indicative of separate populations of distinct chemistry and nanomechanical properties.

On the other hand, Figure 5 shows strong negative correlations of resonant *s*-SNOM phase with adhesion (Figure 5a,b) and dissipation (Figure 5c,d) channels, which are predominantly sensitive to chemical composition. The bivariate histograms (Figure 5a,c) show a distinct bimodal distribution, more apparent than in the histogram of each channel alone (shown in projection onto respective axes), which is further exemplified by summing diagonally across the direction of correlation to create the coincidence histograms shown in Figure 5b and Figure 5d. The two populations in this distribution represent distinct regions with separate chemical and nanomechanical properties.

## Conclusion

In summary, we have combined spatio-spectral *s*-SNOM with force–distance spectroscopy to create a multimodal dataset of material and optical properties of nanoscale heterogeneous soft matter. Using a single AFM/*s*-SNOM setup and a metallized scanning probe tip, we produced images of a particular 500 × 500 nm size region of a PS-*b*-PMMA film via IR *s*-SNOM and force–distance spectroscopy analyzed with computational imaging techniques. These maps are sensitive not only to chemical composition, but also to inhomogeneity within domains arising from, for example, varying degrees of crystallinity. The hybrid combination of different imaging modalities established here promises to be a powerful tool for measuring and understanding intermolecular interactions and could address fundamental questions for the study and design of functional nanomaterials.

## Acknowledgements

We thank Kyoung-Duck Park for assistance in the preliminary stages of these measurements and Omar Khatib and Eric Muller for valuable discussions at various stages of the data analysis. Funding is gratefully acknowledged from the National Science Foundation (CHE 1306398). This work was also supported by the Soft Materials Research Center under NSF MRSEC Grant DMR-1420736.

## References

[1] Muller E A, Pollard B, Raschke M B (2015). J Phys Chem Lett.

[2] Taubner T, Hillenbrand R, Keilmann F (2004). Appl Phys Lett.

[3] Huth F, Govyadinov A, Amarie S, Nuansing W, Keilmann F, Hillenbrand R (2012). Nano Lett.

[4] Bechtel H A, Muller E A, Olmon R L, Martin M C, Raschke M B (2014). Proc Natl Acad Sci U S A.

[5] Berweger S, Nguyen D M, Muller E A, Bechtel H A, Perkins T T, Raschke M B (2013). J Am Chem Soc.

[6] Pollard B, Muller E A, Hinrichs K, Raschke M B (2014). Nat Commun.

[7] Westermeier C, Cernescu A, Amarie S, Liewald C, Keilmann F, Nickel B (2014). Nat Commun.

[8] Amenabar I, Poly S, Nuansing W, Hubrich E H, Govyadinov A A, Huth F, Krutokhvostov R, Zhang L, Knez M, Heberle J (2013). Nat Commun.

[9] Butt H-J, Cappella B, Kappl M (2005). Surf Sci Rep.

[10] Radmacher M, Tillamnn R W, Fritz M, Gaub H E (1992). Science.

[11] Ton-That C, Shard A G, Teare D O H, Bradley R H (2001). Polymer.

[12] Sweers K, van der Werf K, Bennink M, Subramaniam V (2011). Nanoscale Res Lett.

[13] Young T J, Monclus M A, Burnett T L, Broughton W R, Ogin S L, Smith P A (2011). Meas Sci Technol.

[14] Herruzo E T, Perrino A P, Garcia R (2014). Nat Commun.

[15] Francis T J, Vogt B D, Wang M X, Watkins J J (2007). Macromolecules.

[16] Walheim S, Böltau M, Mlynek J, Krausch G, Steiner U (1997). Macromolecules.

[17] Green P F, Christensen T M, Russell T P, Jérôme R (1989). Macromolecules.

[18] Sevink G J A, Zvelindovsky A V, van Vlimmeren B A C, Maurits N M, Fraaije J G E M (1999). J Chem Phys.

[19] Stenbock-Fermor A, Knoll A W, Böker A, Tsarkova L (2014). Macromolecules.

[20] Knoll R, Magerle K, Krausch G (2001). Macromolecules.

[21] Sader J E, Chon J W M, Mulvaney P (1999). Rev Sci Instrum.

[22] Taubner T, Keilmann F, Hillenbrand R (2004). Nano Lett.

[23] Aizpurua J, Taubner T, García de Abajo F J, Brehm M, Hillenbrand R (2008). Opt Express.

[24] Derjaguin B V, Muller V M, Toporov Yu P (1994). Prog Surf Sci.

[25] Humbert S, Lame O, Séguéla R, Vigier G (2011). Polymer.

[26] Briscoe B J, Fiori L, Pelillo E (1999). J Phys D: Appl Phys.

[27] Jee A-Y, Lee M (2010). Polym Test.

[28] Elbs H, Fukunaga K, Stadler R, Sauer G, Magerle R, Krausch G (1999). Macromolecules.

[29] Sirghi L, Nakagiri N, Sugisaki K, Sugimura H, Takai O (2000). Langmuir.

[30] Zitzler L, Herminghaus S, Mugele F (2002). Phys Rev B.

[31] Sirghi L (2012). Langmuir.

[32] Pittenger B, Erina N, Su C (2012). Bruker Application Note.

[33] Russell T P, Coulon G, Deline V R, Miller D C (1989). Macromolecules.

[34] Jung Y C, Bhushan B (2006). Nanotechnology.

[35] Fuchs K, Friedrich C, Weese J (1996). Macromolecules.

